# Protective Roles of Sodium Selenite against Aflatoxin B_1_-Induced Apoptosis of Jejunum in Broilers

**DOI:** 10.3390/ijerph111213130

**Published:** 2014-12-17

**Authors:** Xi Peng, Shengqiang Zhang, Jing Fang, Hengmin Cui, Zhicai Zuo, Junliang Deng

**Affiliations:** Key Laboratory of Animal Diseases and Environmental Hazards of Sichuan Province, College of Veterinary Medicine, Sichuan Agricultural University, Ya’an 625014, China; E-Mails: pengxi197313@163.com (X.P.); 15892685640@163.com (S.Z.); zzcjl@126.com (Z.Z.); dengjl213@126.com (J.D.)

**Keywords:** aflatoxin B_1_, apoptosis, TUNEL, Bcl-2, Bax, Caspase-3

## Abstract

The effects of aflatoxin B_1_ (AFB_1_) exposure and sodium selenite supplementation on cell apoptosis of jejunum in broilers were studied. A total of 240 one-day-old male AA broilers were randomly assigned four dietary treatments containing 0 mg/kg of AFB_1_ (control), 0.3 mg/kg AFB_1_ (AFB_1_), 0.4 mg/kg supplement Se (+ Se) and 0.3 mg/kg AFB_1_ + 0.4 mg/kg supplement Se (AFB_1_ + Se), respectively. Compared with the control broilers, the number of apoptotic cells, the expression of Bax and Caspase-3 mRNA were significantly increased, while the expression of Bcl-2 mRNA and the Bcl-2/Bax ratio were significantly decreased in AFB_1_ broilers. The number of apoptotic cells and the expression of Caspase-3 mRNA in AFB_1_ + Se broilers were significantly higher than those in the control broilers, but significantly lower than those in AFB_1_ broilers. There were no significant changes in the expression of Bax mRNA between AFB_1_ + Se and control broilers; the expression of Bcl-2 mRNA and the Bcl-2/Bax ratio in AFB_1_ + Se broilers were significantly lower than those in the control broilers, but significantly higher than those in AFB_1_ broilers. In conclusion, 0.3 mg/kg AFB_1_ in the diet can increase cell apoptosis, decrease Bcl-2 mRNA expression, and increase of Bax and Caspase-3 mRNA expression in broiler’s jejunum. However, supplementation of dietary sodium selenite at the concentration of 0.4 mg/kg Se may ameliorate AFB_1_-induced apoptosis by increasing Bcl-2 mRNA expression, and decreasing Bax and Caspase-3 mRNA expression.

## 1. Introduction

Aflatoxin B_1_ (AFB_1_) is a well-known mycotoxin produced by different strains of *Aspergillus flavus* and *Aspergillus parasiticum*. In humans and various animal species, it has been reported to be a potent hepatotoxic and hepatocarcinogenic agent [1,2]. AFB_1_ is readily transported across the plasma membrane and interacts with nucleic acids and proteins, altering various cellular activities [3]. Previous research has shown that poultry are extremely sensitive to the toxic and carcinogenic action of AFB_1_, resulting in millions of dollars in annual losses to producers due to reduced growth rates, reduced egg production, increased susceptibility to disease, and other adverse effects [4,5,6,7,8,9]. 

Apoptosis is a specialized process of cell death that is part of the normal development of organs and tissue maintenance, but may also occur as a response to various environmental stimuli, indicating toxicity. Early research has shown that AFB_1_ is able to being a direct and indirect initiator as well as promoter of apoptotic process [10,11]. Several studies indicated that AFB_1_ induced apoptosis of different cells, such as hepatocyte [12], bone marrow cells [13], and bronchial epithelial cells [14]. Moreover, Chen *et al.* [15] reported that 0.3 mg/kg AFB_1_ in the broilers’ diet could induce the increase of apoptotic thymocytes by the up-regulation of Bax and Caspase-3 expression and the down-regulation of Bcl-2 expression. Similarly, Wang *et al.* [16] has demonstrated that AFB_1_ could increase the percentage of apoptotic splenocytes in broilers, which was closely related to oxidative stress. However, the effects of AFB_1_ on the apoptosis of jejunum were rarely reported. The gastrointestinal tract is the main site where conversion and absorption of food components takes place. As part of the small intestine, the jejunum is the major component of the gastrointestinal tract. Epithelium cells in the small intestine have a high turnover and as it is essential to maintain normal balance, apoptosis is crucial for maintenance of normal morphology and function [17,18,19]. Since poultry are extremely sensitive to the toxic and carcinogenic action of AFB_1_, studies on AFB_1_-related apoptosis in the jejunum in broilers are very important.

As an important micronutrient for humans and animals, Selenium (Se) plays a vital role in biological systems, such as chemopreventive [20], antioxidant, detoxification [21] and anticancer effects [22], and effects on both the innate and acquired immune system [23,24]. Furthermore, Se plays a key role in cell apoptosis [22]. At nutritional doses, Se is an essential component of selenocysteine (SeCys) in selenoproteins, and it promotes cell cycle progression and prevents cell death [22]. Previous studies have shown that Se could counteract the adverse effects of AFB_1_ in poultry [4,15,16,24,25]. For example, Se may ameliorate AFB_1_-induced lesions of the thymus and accordingly improve the impaired cellular immune function in broilers [15]. Similarly, Se may exhibit protective effects on AFB_1_-induced splenic toxicity by inhibiting oxidative stress and excessive apoptosis [16]. Recently, our study has demonstrated that supplementation of dietary sodium selenite at the concentration of 0.4 mg/kg Se protected the jejunum from the developmental retardation, decreased proliferation, and G_2_/M phase arrest caused by AFB_1_ [26]. However, the effects of Se against AFB_1_-induced jejunal cell apoptosis have yet not been reported. In the present research, experiments were conducted to examine the effect of AFB_1_ exposure and sodium selenite supplementation on the cell apoptosis of broilers’ jejunum by TUNEL assay and quantitative real-time PCR.

## 2. Materials and Methods

### 2.1. Animals and Diets

Two hundred forty 1-day-old healthy male AA broilers were obtained from a commercial rearing farm (Wenjiang poultry farm, Sichuan Province). Chickens were randomly assigned four dietary treatments containing 0 mg/kg of AFB_1_ (control), 0.3 mg/kg AFB_1_ (AFB_1_), 0.4 mg/kg supplement Se (+ Se) and 0.3 mg/kg AFB_1_ + 0.4 mg/kg supplement Se (AFB_1_ + Se), respectively. Our earlier studies have demonstrated that 0.3 mg/kg AFB_1_ in diet had obvious adverse effects on broilers, and an appropriate level of Se supplied in the diet (0.4 mg/kg) could provide optimal protective effects against AFB_1_-induced toxicity in broilers [15,16]. Based on this information, an appropriate toxin concentration (0.3 mg/kg AFB_1_) and dietary Se level (0.4 mg/kg) were chosen. 1% Feed-grade sodium selenite was mixed into the control diet to formulate + Se and AFB_1_ + Se diets containing 0.4 mg/kg Se supplement by a stepwise dilution method. AFB_1_ was obtained from Pribolab Pte. Ltd (Singapore). The AFB_1_-contaminated diets were made up according to the method described by Kaoud [27]. Briefly, 3 mg AFB_1_ was completely dissolved in 30 mL methanol, and then the 30 mL mixture was mixed into a 10 kg corn-soybean basal diet to formulate the AFB_1_ and AFB_1_ + Se diets, respectively. An equivalent amount of methanol was mixed into corn-soybean basal diet to produce the control diet. Then the methanol of the diets was evaporated at 98 °F (37 °C)(the concentration of dietary AFB_1_ was not detected in this experiment, but it can be assured that any possible back-ground contamination was evenly distributed among the experimental groups throughout the trial because the same lot of basal diet was used for formulating experimental diets). The content of Se (0.332 mg/kg) in the control diet was analyzed by hydride-generation atomic absorption spectroscopy. Broilers were housed in cages with electrically heated units and were provided with water as well as the aforementioned diets *ad libitum* for 21 days. The basal diets were formulated according to National Research Council (NRC, 1994) and Chinese Feeding Standard of Chicken (NY/T33-2004) recommendations to meet the nutrient requirements of broilers from 1 to 21 days. The composition of the basal diets is presented in Table 1. All procedures of the experiment were performed in compliance with the laws and guidelines of Sichuan Agricultural University Animal Care and Use Committee.

### 2.2. Clinical Signs and Body Weight

The clinical symptoms were observed each day. At 7, 14 and 21 days of age during the experiment, the body weight of chicken in each group was measured.

**Table 1:** Composition of the Basal Diet.

Composition	Content (%)	Nutrient	Content (%)
corn	51.95	crude protein (CP)	21.50
soybean	39.50	Methionine (Met)	0.50
rapeseed oil	4.10	calcium (Ca)	1.00
D,L-metionine	0.20	all phosphorus (P)	0.70
calcium hydrogen phosphate	1.85	Methionine + cysteine (Met+Cys)	0.84
calcium carbonate	1.30	lysine (Lys)	1.15
sodium chloride	0.40	Threonine (Thr)	0.83
trace element premix **^a^**	0.50	metabolizable energy (ME) (MJ/Kg)	29.90
choline	0.17		
multivitamins **^b^**	0.03		
total	100		

Note: **^a^** trace element premix (mg/kg): FeSO_4_·7H_2_O, 530; CuSO_4_·5H_2_O, 30; MnSO_4_·H_2_O, 400; ZnSO_4_·7H_2_O, 470; KI, 18; NaSeO_3_, 0.3. **^b^** Multivitamins: Vitamin A, 13500 IU/kg; Vitamin D, 3000 IU/kg; Vitamin E, 24 IU/kg; Vitamin K_3_, 3 mg/kg; pantothenic acid, 15 mg/kg; folic acid,1.05 mg/kg; nicotinamide, 30 mg/kg; biotin, 0.14 mg/kg.

### 2.3. TUNEL Immunohistochemistry

At the end of 7, 14, and 21 days, six chickens in each treatment were euthanized, and the jejunum (the midpoint between the bile duct entry and Meckel’s diverticulum) were immediately fixed in 4% paraformaldehyde. After fixation for 24 h, tissues were dehydrated, paraffin-embedded, sectioned into 5 μm slices. Sections were stained with TUNEL immunohistochemistry assay, which was performed using apoptosis detection kit (QIA33, Merck, Darmstadt, Germany) according to the manufacturer’s instructions, as described by Peng *et al* [28]. The number of TUNEL-positive cells was evaluated in the apical region of villi using Image-Pro Plus 5.1 (Media Cybernetics, Silver Spring, MD, USA) image analysis software. For each sample, five random fields of 0.064 mm^2^ were quantified (corresponding approximately to five fields at ×400 magnification), respectively. Results were expressed as the average of TUNEL-positive cells per 0.064 mm^2^ area. 

### 2.4. Quantitative Real-Time PCR (qRT-PCR)

Quantitative real-time PCR (qRT-PCR) assay was carried out as reported by Chen *et al.* [15]. Briefly, the jejunal mucosae from six chickens in each treatment at 7, 14, and 21 days of the experiment were stored in liquid nitrogen, respectively. Adding liquid nitrogen, the jejunal mucosae were crushed with pestle to homogenize until powdery, respectively. Total RNA was extracted from the powdery of jejunal mucosae using RNAiso Plus (9108/9109, Takara, Otsu, Japan). The mRNA was then reverse transcribed into cDNA using PrimScript^TM^ RT reagent Kit with gDNA Eraser (RR047A, Takara, Otsu, Japan). The cDNA was used as a template for quantitative real-time PCR analysis. 

For qRT-PCR reactions, 25 μL mixtures were made by using SYBR^®^ Premix Ex Taq^TM^ II (DRR820A, Takara , Otsu, Japan), containing 12.5 μL Tli RNaseH Plus, 1.0 μL of forward and 1.0 μL of reverse primer, 8.5 μL RNAase-free water and 2 μL cDNA. Reaction conditions were set to 3 min at 95 °C (first segment, one cycle), 10 s at 95 °C and 30 s at Tm of a specific primer pair (second segment, 44 cycles) followed by 10 s at 95 °C, and 72 °C for 10 s (dissociation curve segment) using Thermal Cycler (C1000, BIO RAD, CA, USA). The mRNA expression of Bax, Bcl-2 and Caspase-3 was analyzed, and β-actin was used as an internal control gene. Sequence of primers was obtained from GenBank of NCBI. Primers were designed with Primer 5, and synthesized by BGI Tech (Shenzhen, China). The oligonucleotides used as primers in RT-qPCR analysis of Bax, Bcl-2, Caspase-3 and β-actin were determined according to the references [29,30,31]. The control broilers responses (mRNA amount) were been as reference values for between treatments comparisons within the same control day in each week, respectively. The results were analyzed with $2^{-\Delta \Delta C_{T}}$ calculation method [32].

### 2.5. Statistical Analysis

The results were shown as means ± standard error (M ± SE). Statistical analyses were performed using one-way analysis of variance, and Dunnett’s test was employed for multiple comparisons. A value of *p* < 0.05 was considered significant.

## 3. Results

### 3.1. Clinical Signs and Body Weight

There were no evident clinical symptoms among four treatments. The body weight of broilers showed no significant differences between treatments at 1, 7, and 14 days of age (*p* > 0.05) (Table 2). At 21 days of age, the body weight in AFB_1_ broilers was significantly lower than that in control broilers (*p* < 0.05), but no significant differences occurred among control, +Se and AFB_1_+Se broilers (*p* > 0.05) (Table 2).

**Table 2:** The Body Weights of Broilers (g).

Time	Treatments			
	Control	AFB_1_	+Se	AFB_1_ + Se
1 day (*n* = 60)	41.56 ± 3.36	41.81 ± 3.23	41.59 ± 3.26	41.86 ± 3.02
7day (*n* = 60)	121.81 ± 7.45	120.66 ± 7.38	120.56 ± 8.77	121.93 ± 6.84
14day (*n* = 42)	328.62 ± 17.36	323.30 ± 22.26	324.51 ± 20.14	327.49 ± 20.70
21day (*n* = 24)	695.71 ± 37.77 **^a^**	672.25 ± 37.95 **^b^**	681.80 ± 26.84	694.44 ± 41.85

Note: data are presented with the means ± standard error. Figures marked with the different small superscript letters are significantly different (**^a,b^** *p* < 0.05) between treatment and control within the time of exposure.

### 3.2. TUNEL Immunohistochemistry

**Figure 1:**
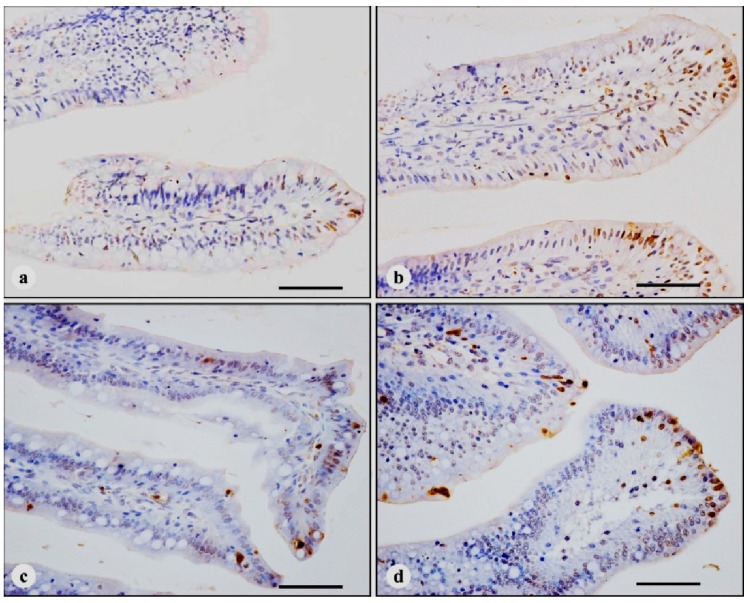
TUNEL-positive cells in the apical regions of jejunal villi at 14 days of age, immunohistochemistry stain. (**a**) in control broiler; (**b**) in AFB_1_ broiler, the number of TUNEL-positive cells is increased, compared with control broiler; (**c**) in + Se broiler, the number of TUNEL-positive cells is not obviously changed compared with control broiler; (**d**) in AFB_1_ + Se broiler, the number of TUNEL-positive cells is increased compared with control broiler, but, decreased compared with AFB_1_ broiler. Bars = 50 μm.

**Figure 2:**
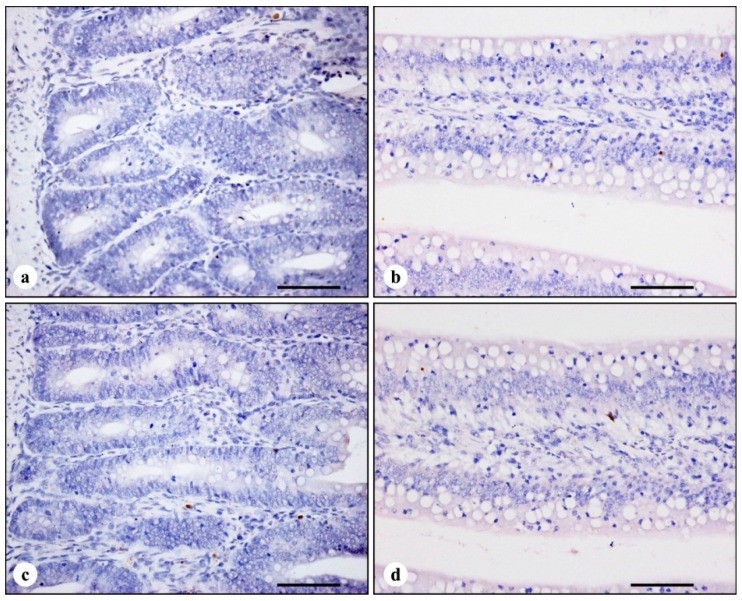
TUNEL immunohistochemistry staining in the crypts and in the middle regions of jejunal villi at 14 days of age. There are a few scattered TUNEL-positive cells in the crypts and in the middle regions of villi in all treatments. (**a**) The crypts in control broiler; (**b**) the middle regions of villi in control broiler; (**c**) the crypts in AFB_1_ broiler; (**d**) the middle regions of villi in AFB_1_ broiler. Bars = 50 μm.

In four treatments, the nuclei of TUNEL-positive cells were stained brown. TUNEL-positive cells were mainly distributed in the apical region of villi (Figure 1), with a few scattered positive cells in the middle and basal regions of villi and the crypt (Figure 2). Compared with control broilers on days 7, 14 and 21, the number of TUNEL-positive cells in AFB_1_ broilers was significantly increased (*p* < 0.01), however, the number of TUNEL positive cells in + Se broilers showed no significant changes (*p* > 0.05). In addition, the number of TUNEL-positive cells in AFB_1_ + Se broilers was significantly higher than that in control broilers (*p* < 0.01), but, significantly lower (*p* < 0.01) than that in AFB_1_ broilers during the experiment. The number of TUNEL-positive cells in the apical region of villi is shown in Table 3.

**Table 3:** The Number of TUNEL-positive Cells in the Apical Region of Jejunal Villi (number/0.064 mm^2^).

Time	Treatments			
	control	AFB_1_	+ Se	AFB_1_ + Se
7 day	5.200 ± 0.227 **^C^**	18.900 ± 0.273 **^A^_B_**	5.533 ± 0.184 **^C^**	11.767 ± 0.243 **^B^_B_**
14 day	5.600 ± 0.212 **^C^**	18.833 ± 0.322 **^A^_B_**	5.267 ± 0.33 **^C^**	13.300 ± 0.437 **^B^_A_**
21 day	5.100 ± 0.175 **^C^**	19.767 ± 0.493 **^A^_A_**	5.567 ± 0.233 **^C^**	12.100 ± 0.175 **^B^_A_**

Note: data are presented with the means ± standard error (*n* = 6). Figures marked with the different capital letters are significantly different (*p* < 0.01). ***^A–C^*** *p* < 0.01 between treatment and control within the time of exposure; **_A,B_** *p* < 0.01 between times of exposure within the control or treatment.

### 3.3. Quantitative Real-Time PCR (qRT-PCR)

The mRNA expressions of Bax, Bcl-2 and Caspase-3 and the Bcl-2/Bax ratio in jejunal mucosa are shown in Table 4. The expressions of Bax and Caspase-3 mRNA in AFB_1_ broilers were significantly higher than those in control broilers at 7, 14 and 21 days of age (*p* < 0.01). There were no significant changes in the expression of Bax mRNA between AFB_1_ + Se broilers and control broilers at 7, 14 and 21 days of age. In addition, the expression of Caspase-3 mRNA in AFB_1_ + Se broilers was significantly higher (*p* < 0.01) than that in control broilers, but significantly lower (*p* < 0.05) than that in AFB_1_ broilers during the experiment, except for at 14 days of age (Table 4). Compared with control broilers, the expression of Bcl-2 mRNA and the Bcl-2/Bax ratio in AFB_1_ broilers significantly decreased on days 7, 14 and 21 (*p* < 0.01). The expression of Bcl-2 mRNA and the Bcl-2/Bax ratio in AFB_1_ + Se broilers were significantly lower than those in control broilers (*p* < 0.01 or *p* < 0.05), but significantly higher than those in AFB_1_ broilers (*p* < 0.01 or *p* < 0.05) (Table 4).

**Table 4:** The mRNA Expression of Bax, Bcl-2 and Caspase-3 and Bcl-2/Bax Ratio in Jejunal Mucosa.

Item	Time	Treatments			
		Control	AFB_1_	+ Se	AFB_1 _+ Se
Bax	7 day	1.003 ± 0.054 **^B^**	1.218 ± 0.028 **^A^_b_**	0.936 ± 0.035 **^B^**	1.024 ± 0.019 **^B^**
	14 day	1.001 ± 0.037 **^B^**	1.235 ± 0.020 **^A^_b_**	0.948 ± 0.019 **^Bb^**	1.040 ± 0.014 **^Ba^_b_**
	21 day	1.003 ± 0.054 **^B^**	1.302 ± 0.026 **^A^_a_**	0.954 ± 0.030 **^B^**	0.993 ± 0.013 **^B^_a_**
Bcl-2	7 day	1.001 ± 0.027 **^Aa^**	0.748 ± 0.009 **^B^**	0.940 ± 0.013 **^Ab^**	0.923 ± 0.014 **^Ab^_a_**
	14 day	1.001 ± 0.027 **^Aa^**	0.770 ± 0.020 **^B^**	0.972 ± 0.025 **^A^**	0.894 ± 0.024 **^Ab^**
	21 day	1.001 ± 0.039 **^A^**	0.757 ± 0.018 **^Bc^**	0.976 ± 0.016 **^ACa^**	0.857 ± 0.031 **^BCb^_b_**
Bcl-2/Bax	7 day	1.001 ± 0.030 **^Aa^**	0.615 ± 0.013 **^B^**	1.007 ± 0.050 **^Aa^**	0.902 ± 0.006 **^Ab^**
	14 day	1.003 ± 0.054 **^Aa^**	0.623 ± 0.008 **^D^**	1.025 ± 0.027 **^AC^**	0.860 ± 0.029 **^ABb^**
	21 day	1.002 ± 0.042 **^Aa^**	0.582 ± 0.026 **^B^**	1.025 ± 0.031 **^Aa^**	0.863 ± 0.042 **^Ab^**
Caspase-3	7 day	1.000 ± 0.016 **^B^**	1.247 ± 0.023 **^Aa^_b_**	1.029 ± 0.027 **^B^**	1.166 ± 0.017 **^Ab^_b_**
	14 day	1.003 ± 0.054 **^C^**	1.302 ± 0.009 **^A^_a_**	1.036 ± 0.050 **^BCb^**	1.195 ± 0.014 **^ABa^**
	21 day	1.002 ± 0.040 **^B^**	1.305 ± 0.010 **^Aa^_a_**	0.995 ± 0.021 **^B^**	1.214 ± 0.016 **^Ab^_a_**

Note: data are presented with the means ± standard error (*n* = 6). The data are expressed as relative responses with respect to the control. Figures marked with the different capital letters are significantly different (*p* < 0.01); figures marked with the different small letters are significantly different (*p* < 0.05). ***^A–D^*** *p* < 0.01 or ***^a–c^*** *p* < 0.05 between treatment and control within the time of exposure; **_a,b_** *p* < 0.05 between times of exposure within the control or treatment.

## 4. Discussions

The results of clinical signs and body weight observed in this study showed that 0.4 mg/kg supplemented dietary Se could be safe for chickens, which is in agreement with Cai *et al*.’s report [33]. AFB_1_ (0.3 mg/kg) did not induce evident clinical symptoms, but significantly decreased body weight of broiler at 21 days of age was observed. It is thus suggested that 0.3 mg/kg AFB_1_ may retard the growth of the broiler. As reported in a review paper, broiler’s performance may be affected, when the concentration of dietary AFB_1_ is about 0.5 mg/kg [34].

Tissue homeostasis depends on both cell proliferation and cell death. The small intestinal epithelium is a rapidly renewing tissue, in which cells are lost from the villus into the gut lumen and are generally replaced at an equal rate by the proliferation of cells in the crypts [18]. Early researches indicate that apoptosis is responsible for controlling the majority of intestinal epithelial cell loss, and apoptosis is occurs predominantly in the villus tip cells [18], which is supported by the following observation (1) high levels of the pro-apoptotic protein, Bax, have been detected in these terminally differentiated cells [18]; (2) the expression of a possible apoptotic endonuclease (DNase I) also increases towards the villus tip [35]; (3) possible increased expression of transforming growth factor-β and evidence for reduced adhesion may also lend support to this hypothesis [36,37,38]; (4) The presence of large numbers of macrophages and lymphocytes at the villus tip is consistent with apoptosis of terminally differentiated cells [39]. TUNEL assay can identify DNA fragmentation and examine the topographic distribution of apoptotic cells [15,40,41]. Similar to previous reports in other animals’ intestine [42,43], in the present study, TUNEL-positive cells in all treatments were predominantly distributed in the apical region of the villus. Therefore, TUNEL-positive cells in the villi tip were counted as the number of apoptotic cells in the jejunal mucosa.

Several studies have indicated that AFB_1_ was able to induce apoptosis in hepatocytes, lung and bone marrow cells, bronchial epithelial cells, thymocytes and splenocytes [12,13,14,15,16]. Our result shows that the number of TUNEL-positive cells was increased in AFB_1_ broilers when compared with control broilers. This result indicates that AFB_1_ could induce excessive apoptosis of broilers’ jejunum. Our early study has revealed that 0.3 mg/kg AFB_1_ in the diet can induce pathological lesions (shedding) and reduce cellular proliferation of broilers’ jejunum [26]. Epithelial cells of the intestine experience permanent renewal that includes cell proliferation, migration, differentiation, apoptosis, and cell shedding into the intestinal lumen [18,44]. Homeostasis of these activities is essential for structural and functional properties of intestine [45,46]. Also, both decreased proliferation and/or increased cell death may reduce cell number, whereas increased proliferation and/or decreased death may increase cell number [18]. Therefore, the increased apoptosis observed in this study, the decreased proliferation [26] and pathological shedding in the villus tips in broilers’ jejunum induced by AFB_1_ [26] may lead to the decreased enterocytes which may be followed by the declined function of this organ. 

Previous researches have demonstrated that AFB_1_ could lead to cellular apoptosis via mitochondrial or cell death receptor pathways [47,48,49,50]. In the present study, the mRNA expression of Bax, Bcl-2 and Caspase-3 was determined for evaluating whether AFB_1_-induced apoptosis of jejunum is related to the mitochondrial pathway. The results showed that the mRNA expression of Bax and Caspase-3 significantly increased, while the mRNA expression of Bcl-2 and the ratio of Bcl-2/Bax significantly decreased in AFB_1_ broilers, when compared with those in control broilers. These results suggest that the excessive apoptosis of jejunum induced by AFB_1_ was onset by the mitochondrial signaling pathway, which is accord with the previous research in human bronchial epithelial cells [14] and broiler thymocytes [15]. Future studies should focus on whether AFB1-induced apoptosis in jejunal cells is triggered by a cell death receptor pathway. Previous studies have revealed that AFB_1_ might induce oxidative stress by the formation of Reactive Oxygen Species (ROS) and the decrease of the activity and the gene expression of antioxidant enzymes [51,52,53,54,55,56]. As an important physiological effector of apoptosis [57], ROS induces the disruption of the mitochondrial membrane potential (MMP) and formation of mitochondrial apoptosis-induced channel promoted by Bax, by which cytochrome c releases from mitochondria [58]. Cytochrome c compounds in cytoplasm can activate caspase-9 followed by activation of Caspase-3 promoting the apoptosis process [59]. 

In the present study, the number of apoptotic cells had no significant difference between + Se and control broilers from 7 to 21 days of age. In addition, no significant difference was observed in the ratio of Bcl-2/Bax between + Se and control broilers during the experiment. The result suggests that 0.4 mg/kg Se supplied in the diet had almost no obvious effects on the apoptosis of broilers’ jejunum.

Recent studies have shown that Se has protective action against cell apoptosis induced by AFB_1_ in poultry [15,16]. In the present research, the number of apoptotic cells and the expression of Caspase-3 mRNA in AFB_1_ + Se broilers were significantly lower than those in AFB_1_ broilers during the experiment, and the expression of Bcl-2 mRNA and the Bcl-2/Bax ratio was significantly higher than those in AFB_1_ broilers. These results indicate that the diet supplemented with 0.4 mg/kg Se might have protective roles against AFB_1_-induced jejunal apoptosis of broiler by the upregulation of the Bcl-2/Bax ratio. Similar results were also reported in broilers’s thymus and spleen by Chen and Wang [15,16]. Previous researches showed that AFB_1_-induced apoptosis could be caused by lipid peroxidation and oxidative DNA damage [60,61]. However, selenium could repress ROS-mediate apoptosis by inhibiting the apoptosis due to ROS and mitochondrial dysfunction [62,63], which could be related to the antioxidant effects of Se [64]. Our results suggest that appropriate dietary Se could inhibit AFB_1_-induced apoptosis, which may be related to its anti-oxidant function. In comparison with those in the control group, the Bcl-2 expression and the Bcl-2/Bax ratio were significantly decreased, but the Bax expression showed substantially the same extent. This result suggested that supplemented Se could protect AFB_1_-induced apoptosis in some extent, but cannot restore it to the normal level as in the control group, because the Bcl-2 family member can be activated or suppressed by complex factors [65].The mechanism(s) of this observed action require further investigation. 

## 5. Conclusions

In conclusion, 0.3 mg/kg AFB_1_ in the diet can induce an increase of cell apoptosis, a decrease of Bcl-2 mRNA expression, and an increase of Bax and Caspase-3 mRNA expression in broilers’ jejunum. However, supplementation of dietary sodium selenite at the concentration of 0.4 mg/kg Se may ameliorate AFB_1_-induced apoptosis by increasing Bcl-2 mRNA expression, and decreasing Bax and Caspase-3 mRNA expression.
